# Coupling Waveguide-Based Micro-Sensors and Spectral Multivariate Analysis to Improve Spray Deposit Characterization in Agriculture

**DOI:** 10.3390/s19194168

**Published:** 2019-09-26

**Authors:** Anis Taleb Bendiab, Maxime Ryckewaert, Daphné Heran, Raphaël Escalier, Raphaël K. Kribich, Caroline Vigreux, Ryad Bendoula

**Affiliations:** 1 ITAP, Irstea, Montpellier SupAgro, Université de Montpellier, 361 rue Jean-François Breton, 34000 Montpellier, France; 2 ICGM, UMR 5253, cc1503, Université de Montpellier, Place Eugène Bataillon, CEDEX 5, 34095 Montpellier, France; 3 IES, UMR 5214, cc05005, Université de Montpellier, Bâtiment 5, 860 rue Saint-Priest, 34090 Montpellier, France

**Keywords:** optical micro-sensors, crop protection, precision agriculture, infrared spectroscopy, principal component analysis (PCA), partial least squares (PLS), droplet characterization

## Abstract

The leaf coverage surface is a key measurement of the spraying process to maximize spray efficiency. To determine leaf coverage surface, the development of optical micro-sensors that, coupled with a multivariate spectral analysis, will be able to measure the volume of the droplets deposited on their surface is proposed. Rib optical waveguides based on Ge-Se-Te chalcogenide films were manufactured and their light transmission was studied as a response to the deposition of demineralized water droplets on their surface. The measurements were performed using a dedicated spectrophotometric bench to record the transmission spectra at the output of the waveguides, before (reference) and after drop deposition, in the wavelength range between 1200 and 2000 nm. The presence of a hollow at 1450 nm in the relative transmission spectra has been recorded. This corresponds to the first overtone of the O–H stretching vibration in water. This result tends to show that the optical intensity decrease observed after droplet deposition is partly due to absorption by water of the light energy carried by the guided mode evanescent field. The probe based on Ge-Se-Te rib optical waveguides is thus sensitive throughout the whole range of volumes studied, i.e., from 0.1 to 2.5 μL. Principal Component Analysis and Partial Least Square as multivariate techniques then allowed the analysis of the statistics of the measurements and the predictive character of the transmission spectra. It confirmed the sensitivity of the measurement system to the water absorption, and the predictive model allowed the prediction of droplet volumes on an independent set of measurements, with a correlation of 66.5% and a precision of 0.39 μL.

## 1. Introduction

Despite the various campaigns conducted to initiate the reduction of pesticides in agriculture, their use has further increased in recent years [1]. The main challenge is to reduce the use of products while improving plant protection. This challenge motivates numerous research projects that aim to find alternative solutions [2] or to develop decision support tools to reduce doses of applied pesticides [3,4]. Meeting this challenge could significantly reduce the environmental risks associated with agricultural activities. Improving spray efficiency can be a solution implemented to optimize plant protection use in the fields. However, the efficiency of the treatments depends largely on the spray drift which must be minimized, and on the foliar coverage, which must be as large as possible.

Different authors have addressed the spray drift. Examples include the work of Grella et al. [5] who have set up a 20 m-long device, with Petri dishes spaced every 0.5 m. The analysis of the different Petri dishes after spraying with water, in which a yellow marker (E-302 Tartrazine yellow dye tracer) was added, allowed them to compare different spray devices and to highlight those that generate the least important drift. To minimize spray drift, knowledge of droplet size is of paramount importance. Indeed, studies have shown, for example, that the use of sprayers distributing too fine drops leads to a greater drift of the spray and therefore reduces its effectiveness [6]. Techniques to measure the size of the spray droplets can therefore be extremely useful. Different methods already exist. Balsari et al. [7] used, for example, a laser diffraction system (Malvern SprayTec instrument) coupled with a SprayTec software to obtain droplet size data from different sprayers. We can also cite the PDPA (Phase Doppler Particle Analyze) method for example [8]. Other optical methods include the use of a non-contact optical sensor to detect and measure droplets in flight [9] or interferometric techniques (IPI: Interferometric Particle Imaging) [10].

The characterization of leaf coverage surface has also interested researchers. The techniques used were mostly based on water-sensitive papers (WSP) [11], more recently coupled with imaging [12,13,14]. In Reference [14], the authors propose a comparative study of three methods for using data collected on sensitive paper: visual assessment of coverage rate, visual droplet counting and measurement by an imaging system. The correlation obtained was very good only for coverage rates below 20%. In addition to techniques using water-sensitive papers, other methods imply the use of fluorescent tracers. We can mention the work of Llop et al. [15] in which the use of a fluorescent tracer (Helios SC 500; 0.1% *v/v*) traced the deposition on the leaves of the plants (in ng per cm^2^) as well as the coverage rate of the leaves by the sprayed product. In Reference [16], the authors tried to correlate the surface coverage rate and droplet size distribution. To do this, they simultaneously sprayed sensitive papers and Petri dishes containing silicone oil with water to which a red Ponceau marker (with a concentration of 2%) had been added.

As existing qualitative analysis methods adopting the use of sensitive paper or tracers are often not “smart” enough to achieve rapid collection with a single click and automatically adapt to the environment (e.g., changing lighting), researchers have recently proposed an intelligent node based on a vision sensor to automatically collect images of droplet deposition [17]. Other authors have considered the use of a commercial leaf wetness sensor to monitor spraying and have compared the results obtained with the WSP technique [18].

The final objective of our work is to design low-cost micro-sensors that can be used directly in the field to quickly measure leaf coverage surface. It should be noted that our measurement of the coverage surface is an indirect measurement, since we intend to determine the covered surface via a measurement of the volume of droplets deposited on the sensor. Some authors have already proposed such a solution, consisting of an electronic detection system based on resistance [19,20]. However, there is not yet a good enough resolution to detect fine drops. In our case, we propose an integrated optics solution. In a previous work, we fabricated straight waveguides based on Ge-Se-Te chalcogenide films and we tested their sensitivity to water droplet deposition at the wavelength of 1.55 μm. As expected, water absorbed part of the evanescent wave of the guided light, leading to a decrease in the transmitted intensity. Moreover, we showed that the greater the droplet volume and the greater the number of drops, the greater the decrease in intensity [21]. Nevertheless, we observed a non-linearity of the results, with a lower sensitivity for large volumes. Moreover, we limited our study to volumes comprised between 2 and 10 μL, with a step of 2 μL. In this article, we propose to go a little further, even if we are still at the beginning of the development of sensors. The goal was to develop a probe able to measure smaller drops. The studied volumes ranged from 0.1 to 2.5 μL, and a step of 0.1 μL was chosen to estimate the probe precision. Furthermore, we propose, with the same straight waveguides as in our previous work [21], to no longer work at a single wavelength but to use spectroscopy, which, coupled with multivariate analysis, should allow us to increase sensitivity and to consider accessing a measurement of the composition of the sprayed liquid. Among the different methods of multivariate analysis, principal component analysis (PCA) and partial least squares (PLS) techniques, that are commonly used in chemometrics [22], were chosen.

## 2. Materials and Methods

### 2.1. Waveguide Manufacture and Characterization

(a) Manufacture

According to previous studies [21,23,24,25], rib-type waveguides were fabricated. The waveguiding structures were made from a 6 μm-thick Ge_25_Se_65_Te_10_ cladding layer (which refractive index n is 2.55 at the wavelength λ = 1.55 μm) deposited onto silicon substrates and a 6 μm-thick Ge_25_Se_55_Te_20_ core layer (n = 2.65 at λ = 1.55 μm) on top, the core layer being then lithographed, and unprotected parts etched down to 4 μm. Ge-Se-Te films were deposited by thermal co-evaporation, using a MEB 500 device from PLASSYS, Marolles-en-Hurepoix, France. Two current-induced heated sources were used to evaporate Tellurium and Selenium and an electron beam evaporator was used for Germanium. The three sources were placed in a specific configuration to obtain homogeneous films on an area of 4 cm in diameter. Silicon substrates of 3 cm × 4 cm were used to fabricate the components and microscope slides were used for further film characterization. Before deposition, the co-evaporation chamber was evacuated down to 10^−5^ Pa to avoid contamination. During deposition, the substrate holder was rotated for a best homogenization and heated at 70 °C thanks to a hot water circulation. The elemental deposition rates were controlled thanks to three independent thin film deposition controllers (from TELEMARK, Battle Ground, WA, USA and INFICON, Bad Ragaz, Switzerland). The deposition process was initiated when the three elemental deposition rates were stable and was stopped when a total thickness of 6 μm was reached for each film. 

Once the cladding layer and the core layer were deposited onto silicon substrates, the geometry of the core layer was modified by photolithography and ion beam etching, successively. For the photolithographic step, the AZ4533 positive resin and the AZ726 developer from MicroChemicals (Ulm, Germany) were used and samples were insolated during 7 s under a 400 W-UV lamp, through a mask patterned with opaque bands of different widths (ranging from 4 to 42 μm). For the etching step, performed using a set-up provided by PLASSYS (Marolles-en-Hurepoix, France), a mixture of argon and few percent of oxygen was used. The working pressure was fixed to 4.5 × 10^−2^ Pa, the partial pressure P_O2_ being first set to 7.7 × 10^−3^ Pa. A voltage of 400 V was applied, and an etching duration of 50 min was chosen to achieve an etching depth of 4 μm [21].

(b) Characterization

After deposition, each film was analyzed in terms of thickness, composition, and refractive index thanks to a DEKTAK mechanical profilometer (BRUKER, Billerica, MA, USA), a S4500 Energy-Dispersive X-ray spectrometer (HITASHI, Tokyo, Japan) and by exploiting NIR spectra recorded in the range 500–2500 nm using a Cary 5000 spectrophotometer (AGILENT TECHNOLOGIES, Santa Clara, CA, USA), respectively. After etching, the etching depth was checked by profilometry and the component was observed from above and on the edge by using an Inspect S50 scanning electron microscope (FEI, Hillsboro, OR, USA).

### 2.2. Optical Setup 

**Figure 1:**
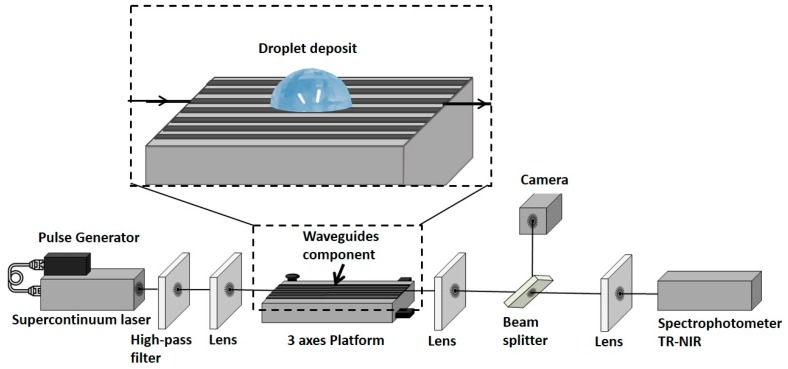
Spectrophotometric bench used to characterize droplet deposits onto the rib waveguide surface. Zoom on the component to visualize the droplet deposited on the top to perform the tests.

The fabricated straight waveguides provided either a multi-mode or a single-mode propagation behaviour, depending on their width and on the wavelength. The bench used for their characterization was equipped with a supercontinuum laser source (SM-100 NIR; LEUKOS, Limoges, France) combined with a pulse generator (4422; SEFRAM, Saint-Etienne, France) (Figure 1). A wavelength high pass filter (FELH1150nm; THORLABS, Newton, NJ, USA) was installed to fit to the Ge-Se-Te waveguides transmission range. To minimize the coupling losses, we used a 3.5 mm focal length lens (87–127 AR; Edmund Optics, Barrington, NJ, USA) to focus the signal from the supercontinuum fiber at the waveguide entrance with a 0.4 numerical aperture. The waveguide sample was held on a 3 axes translation stage platform (M-562; Newport) and fixed by a vacuum mount (HWV001, THORLABS, Newton, NJ, USA). Moreover, we used a convex lens (AL1815-C; THORLABS, Newton, NJ, USA) placed at 11 mm from the output of the waveguide sample to collimate the output beam. This beam hit a beam splitter (BSF10-C, THORLABS, Newton, NJ, USA), 10% of the beam was reflected onto a camera (WDY SMR S320-VS; NIT, Verrières le Buisson, France) to control the injection in the right RIB waveguide. The remaining 90% of the beam was transmitted to a lens (AL1225 M-C; THORLABS, Newton, NJ, USA) to inject light into the spectrometer (UV-VIS-NIR TF; ARCoptix, Neuchatel, Switzerland). This spectrometer performed a Fourier-Transform scanning in the NIR spectrum range (FT-NIR). The measurement spectral range was between 1200 and 2000 nm.

### 2.3. Droplet Deposition and Spectra Acquisition

The influence of the droplet deposition on the waveguide transmission was studied by recording the output spectrum before and after the droplet deposition onto the surface of a rib waveguide. It should be noted that a multi-mode (and therefore wide) guide ensuring the highest possible light intensity collection was selected for the experiments. The temperature of the room was regulated at 24 ± 1 °C. Demineralized water was used in all the experiments and different volumes of droplets were tested. A micropipette was used to dispense droplets from 0.1 to 2.5 μL in volume, in steps of 0.1 μL, on the surface of the rib waveguides. Between two droplet depositions, the sample was dried with dry air and the initial transmission of the waveguide was ensured not having been altered. All the experiments were performed three times on three different days. Each series, corresponding to each day, contains 25 spectra for deposit volumes from 0.1 to 2.5 μL. In total, 75 spectra were thus recorded. The series are called S1, S2 and S3.

For each spectrum, the transmittance T (λ) was calculated thanks to Equation (1) where I_0_ is the spectrum recorded before the droplet deposit, I the spectrum after the droplet deposit and I_n_ the black spectrum (recorded when the shutter of the spectrometer is closed and no light can enter it):

(1) $$T\left(\lambda \right)=\frac{I\left(\lambda \right){-I}_{n}\left(\lambda \right)}{I_{0}\left(\lambda \right){-I}_{n}\left(\lambda \right)},$$

### 2.4. Multivariate Data Analysis

(a) Principal Component Analysis (PCA)

The objective of the multivariate data analysis, in spectroscopy, is to extract information from the different spectra by projecting results in a reduced basis containing much less dimensions than a spectrum acquisition points number (number of wavelengths) [22]. The hypothesis which is done is that this information will be obtained by studying the differences between the spectra. Ideally, spectra are expected to be identical for equal droplet volumes, and on the contrary, the spectra corresponding to the smallest volumes are expected to be a little different from the spectra obtained for the largest volumes. But several parameters can interfere with the measurement, such as the position of the droplet on the waveguide, the temperature or the light injection efficiency; indeed, this latter can vary due to misalignment between series of similar experiments or to laser sources fluctuations between reference (I_0_) and measured (I) spectra; this makes it difficult to exploit the raw spectra. One of the objectives of the PCA will first be to identify the outliers, corresponding to aberrant or atypical spectra to clean the dataset. Another objective will consist in highlighting the differences or similarities between the spectra.

**Figure 2:**
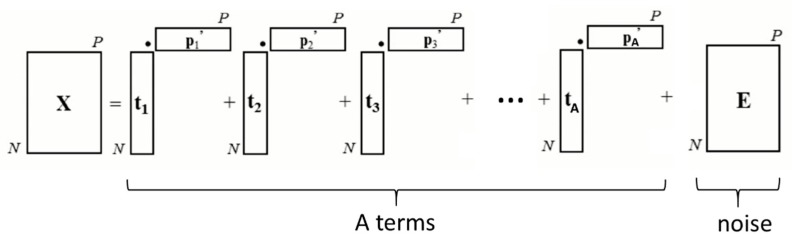
Writing of the matrix X of the initial data according to the PCA model.

PCA consists in trying to define new variables, which will cause as little information loss as possible. Several formalisms can be used, such as matrix writing [26]. Thus, we can write the matrix X of the initial data (a matrix of dimensions *N* × *P*, *N* corresponding to observations–light transmission and P to variables–wavelengths), as the sum of A terms (A being the dimensionality of the new basis, lower than *N* and *P*), which explains most of the data and a residual matrix E corresponding to “noise” (part of the data that are not explained by the analysis). Each of the A terms is itself the product of two vectors t_i_ and p_i_^’^, so that the matrix X of the initial data can be written as in Figure 2:

The *t_i_* are called score vectors, whereas the *p_i_* are called loading vectors [26]. The p_i_ are the coordinates of a principal component (PC) in the original space (the wavelengths space, i.e., the PC is a spectrum) while the *t_i_* are the coordinates of measured spectra in the PC basis. The *p_1_* are computed to maximize the variance of measured spectra versus PC1, and so on for the rest of the variance versus the other PCi. Thus, the PCi are ordered in decreasing percentage of explained variance versus the different recorded spectra, PC1 corresponding to the highest percentage. Usually, the score plot of the first two score vectors *t_1_* and *t_2_,* which thus capture the essential information from the spectra, will highlight similarities or differences between the initial spectra, and will show outliers.

(b) Regression Model

In chemometrics, partial least squares (PLS) regression is the most common regression method for multivariate data [27]. In spectroscopy, PLS is used in most cases for relating spectral data to measured variables [28]. In this study, PLS regression was used to model droplet volume using T(λ). A general PLS model was built using S1 and S2 calibration sets (50 samples) to predict the samples of the independent test set S3 (25 samples).

Before preprocessing, the data acquired in transmittance were transformed in absorbance [−log T(λ)]. The remained spectra were smoothed by using a Savitzky–Golay algorithm [29] with a second order polynomial and 15 points. Then, standard normal variate (SNV) [30] was applied to remove the baseline. The number of latent variables was determined by comparing performances by leave-one-out cross-validation [31].

The performances of the cross-validations (using S1 and S2 sets), and prediction (using the independent set S3) were assessed through the number of latent variables used in the models, the coefficient of determination R^2^ and the standard error of cross-validation (SECV) and standard error of prediction (SEP) corrected from the bias.

All the computations were performed with Matlab software (Matlab R2015b, Mathworks).

## 3. Results & Discussion

### 3.1. Characterization of the Component

Table 1 gives the characteristics of the two deposited layers. The thicknesses errors are less than 2.5%, the compositions are little different (higher atomic percentages in Te but lower atomic percentages in Se) with those expected and the refractive indices n deduced from the transmission spectra are little lower than the targets but the index difference Δn remains around 0.1, as wanted.

**Figure 3:**
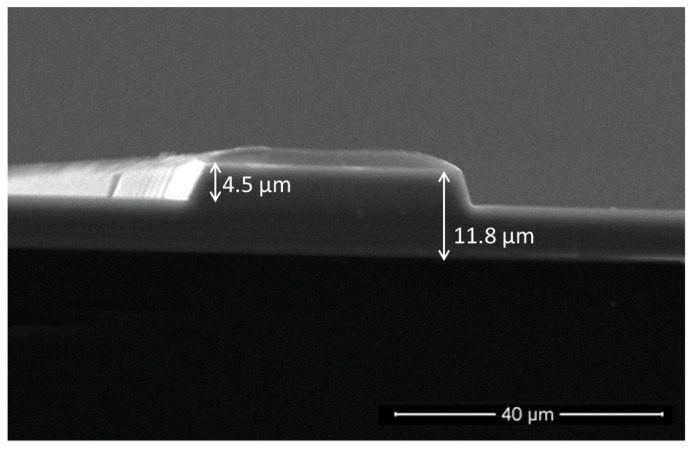
SEM picture of a typical rib waveguide.

Figure 3 shows the typical section of a rib waveguide and allows to confirm the etching depth of 4.5 μm measured by profilometry. This value is close to that expected after an etching of 50 min. A thin layer of resin can be seen, since the images were recorded before the step of removing the excess resin that had not been attacked during etching. Note that the 35 μm-wide rib waveguide presented in Figure 3 is the one used for the experiments.

### 3.2. Raw Spectra

**Figure 4:**
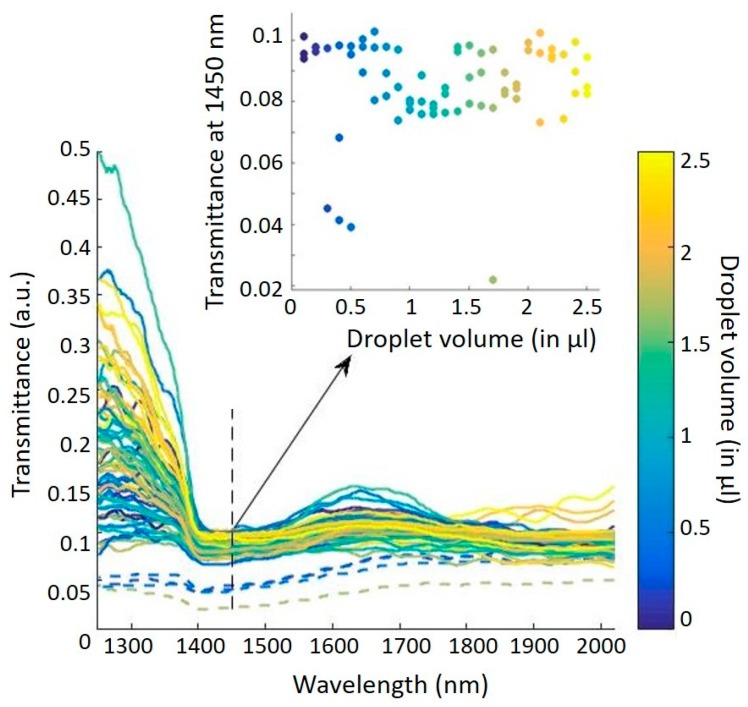
Transmittance spectra obtained from measurements. Outliers are highlighted in dashed lines. The insert shows the transmittance at 1450 nm for each volume.

A total of 75 relative transmission spectra (corresponding to the three series S1, S2, and S3) were recorded and are superimposed in Figure 4. The first remark is that the raw spectra are not noisy, even for low transmissions, which means that the electronic noise is negligible. The second remark is that there is a certain homogeneity in the spectra with the presence of a hollow at about 1450 nm, corresponding to an overtone of the absorption band related to the vibrational energy of the oxygen-hydrogen (O–H) bond in water. This means that our waveguide is sensitive throughout the whole range of volumes studied, i.e., from 0.1 to 2.5 μL, whereas resistance-based sensors did not reach the below 5 μL region [19,20]. However, one can notice that spectra are not gathered depending on the volume: This lack of repeatability is probably due to random fluctuations of the optical alignment. Moreover, four spectra are a little different, with a smaller amplitude in the transmittance, and correspond to the rejected outliers (dashed spectra in Figure 4). To finish, we can see that it is impossible from these raw spectra to extract a correlation between transmittance and volumes. This observation is also true if we focus on the water absorption wavelength (1450 nm), as shown in the insert in Figure 4. The non-correlation between the transmittance of the raw spectra and the droplet volumes justifies the use of a multivariate analysis to solve this problem.

### 3.3. Principal Component Analysis

A principal component analysis (PCA) was performed on the whole dataset. It allowed identifying two principal components PC1 and PC2, explaining 95.81% of the variance between the different recorded spectra (85.72% for PC1 and 10.09% for PC2). We can therefore consider that the main information is contained in these two principal components, the rest can be considered as noise.

**Figure 5:**
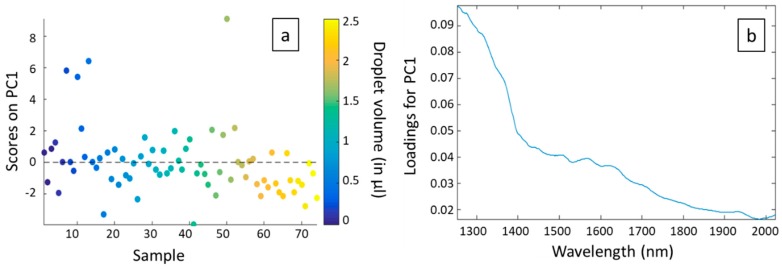
Scores (**a**) and loadings (**b**) related to principal component 1 (PC1). Note that in (a) the samples are classified by increasing volume, regardless of the origin of the series (S1, S2, or S3).

**Figure 6:**
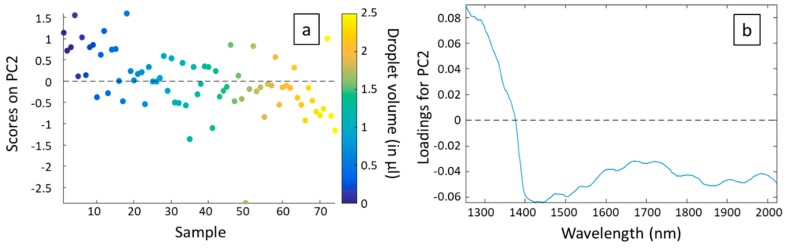
Scores (**a**) and loadings (**b**) related to PC2. Note that in (a) the samples are classified by increasing volume, regardless of the origin of the series (S1, S2, or S3).

**Figure 7:**
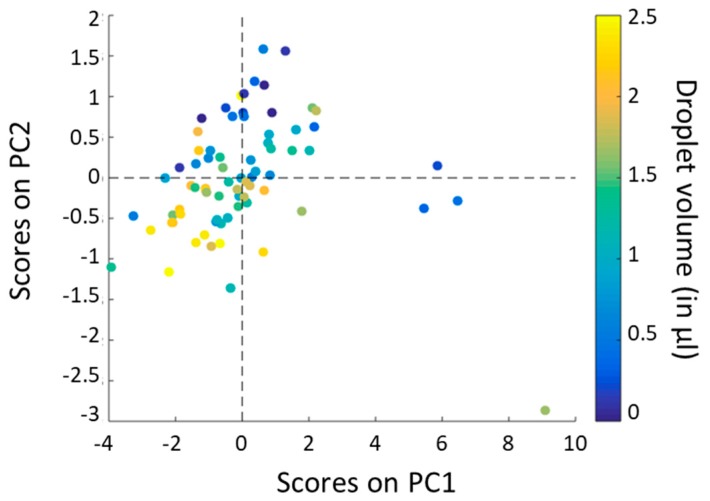
Score plot on PC1 and PC2 generated from the whole dataset.

To go further in the analysis, we plotted the scores on each principal component for each sample, corresponding to the different volumes and measurement days (3 times 25 different volumes). Note that scores represent new coordinates of the different samples on the generated components (Figure 5a for PC1 and Figure 6a for PC2). We also plotted the loadings versus wavelength for each principal component (Figure 5b for PC1 and Figure 6b for PC2). To end, we realized a score plot on PC1 and PC2 generated from the whole dataset (Figure 7).

In Figure 5a we can observe that, except for four points, scores on PC1 are homogeneously distributed around the axis corresponding to a score of 0. It signifies that there is no correlation between PC1 and the droplet volume (color coded). The main variance between the different spectra is thus not due to absorption of water but more to physical phenomena. This is confirmed by Figure 5b. Indeed, we can see that loadings for PC1 are all positive and decrease with the wavelength: PC1 is probably correlated with injection losses (which are supposed to be the lowest at short wavelengths where they were optimized) and propagation losses. Concerning the four points that are in margin of Figure 5a, they correspond to the atypical spectra already observed in Figure 4. They probably correspond to errors in the measurements.

In Figure 6a, we observe a correlation between the scores on PC2 and the droplet volumes. Indeed, even if the effect is not very pronounced, we can notice that scores on PC2 are mainly positive for low volumes, decrease when volumes increase and are mainly negative for high volumes. The principal component PC2 is thus related to the absorption of water. This is confirmed by Figure 6b. Indeed, the loadings for PC2 versus the wavelength form a curve which is totally in agreement with the absorption spectrum of water, with a high absorption peak at around 1450 nm and another one at around 1940 nm. The loadings for PC2 are negative in the spectral range where water absorbs.

Note that the outliers observed in Figure 5a are not observed in Figure 6a. It confirms the fact that these outliers are well correlated to a problem in the measurements and not to the volume of the droplets.

The conclusions that can be drawn from Figure 4 and Figure 5 are therefore that 85.72% of the explained variance between the different spectra is due to physical phenomena, while only 10.09% of the explained variance is due to the presence of water and therefore to the volume of the droplet deposited. Unfortunately, this means that system disturbances have a greater impact on spectra than the measurand. But the positive point is that the effect of volume is measurable.

In Figure 7, corresponding to the score plot on PC1 and PC2, outliers are still present: This is consistent with the previous observations (Figure 4 and Figure 5a). The four spectra corresponding to the four outliers are removed for the prediction model, because a problem of measurement is suspected. Then, we notice that there are no groups formed by the different points (representing the initial spectra): The distribution of the points is indeed quite homogeneous as volume correlated groups overlap. Nevertheless, we find the same trend in PC2 as in Figure 6a: One can see that points corresponding to low volumes are mainly characterized by positive scores whereas points corresponding to high volumes are mainly characterized by negative scores.

### 3.4. Prediction Model

**Figure 8:**
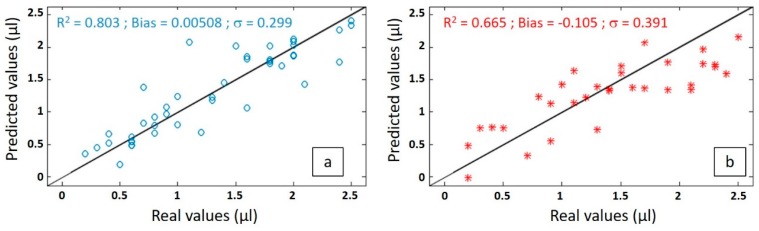
Model performance for the (**a**) cross-validation and (**b**) prediction.

A PLS model was built with a calibration set containing spectral dataset from S1 + S2, after having removed the outliers. The results of the calibration model are presented in Figure 8a where the predicted volumes obtained are plotted versus the real values. The metrological performance of the system (laser + probe + detector + model) can then be extracted. Repeatability is given by the standard error of cross-validation (SECV) or the variance, accuracy by the bias, and precision by the coefficient of correlation R². This latter is equal to 0.803, the bias is 0.00508, and the variance is of 0.299 μL. So, it tends to show that the precision of the model for the calibration set is mainly limited by the variance, since most of it is due to physical perturbation (PC1) and limits the learning process. The low value of the bias may be interpreted as the fact that variance of S1 + S2 is symmetric and has little impact on mean error. The result is quite encouraging.

In order to validate the predictive model in real conditions, it was then tested on the independent spectral dataset from the series S3. The predicted volumes obtained are plotted versus the actual volumes in Figure 8b. The coefficient of correlation is now of 0.665 and the standard error of prediction or the variance in the predicted volume increased from 0.299 to 0.391 μL. The bias is now 50 times much stronger (at a value of −0.105), which negatively impacts the R².

The failure of the coefficient of determination (66.5%) isn’t due to the volume but to the test conditions: Light injection quality, droplet position on the waveguide, spectrometer setting, etc. A first physical improvement is related to light injection, this can be optimized and made robust versus wavelength changes and mechanical vibrations by studying injection alignment impact, pigtailing the fibre from the light source with the waveguide and monitoring directly the output signal of the light source. Also, the temperature may slightly modify the evanescent field extent and thermal monitoring can quantify the temperature explained variance. Finally, droplet lateral position modifies the surface of interaction between water and light and thus varies the output signal; this effect can be studied by imaging droplet position and can be limited by measuring the transmission of a series of close (few microns or tens of microns distance) waveguides on which the droplet is deposited. At the end, by decreasing the physical variance, the percentage of chemically explained variance will increase and improve the learning process to order to obtain a better model. Moreover, the model can be improved from a numerical point of view. Using a bigger calibration test should improve the learning process and, secondly, using a bigger test set should permit better evaluation performance in real conditions. The PLS construction depends on PLS dimension and its error can be minimized by choosing the proper dimensionality.

The 66.5% prediction coefficient of determination can thus be increased, as well as the 0.39 μL precision can be reduced. Nevertheless, it already unveils the possibility of reaching μL or lower resolution, which is more than needed for spray deposit analysis since a less-than-1 μL droplet cannot be deposited as it is suspended in air. The integrated optics approach will permit the addition of functions as power splitting to simplify pigtailing on any number of waveguides in a compact way. Mach–Zehnder or multimode interferometers can be used for on chip temperature monitoring. Additionally, wavelength filters and (de)multiplexers can be used to select wavelengths of interest to reduce the size, complexity, and cost of the system; before this, a study of the reduction of the number of wavelength variables study must be conducted with PCA to feed PLS construction and choose the correct wavelengths depending on prediction performance.

## 4. Conclusions

The accurate determination of the leaf coverage, a key measurement of the spraying process to maximize spray efficiency, requires specific measuring tools. We have developed the first brick of micro-sensors that can be used directly in the field to quickly and automatically measure leaf coverage surface. Even if this work is still a proof of principle, the first results are encouraging. The probe based on Ge-Se-Te rib optical waveguides is sensitive throughout the whole range of volumes studied, i.e., from 0.1 to 2.5 μL.

A principal component analysis performed thanks to spectral measurements showed that the spectra variance was related at 10.09% to the presence of water onto the waveguide surface and that 85.72% of the variance had to be suppressed or strongly limited. The tests for each volume were repeated three times to apply a prediction model that estimates the droplet volume present on the waveguide. First results show that the prediction, on an independent data set, is 66.5% reliable and the precision on the predicted volume is equal to 0.39 μL.

Two complementary ways can be investigated to increase the percentage of variance explained by the volume of the deposited droplets: (a) To increase the measuring system robustness versus interfering physical quantities, and (b) to deepen data analysis with thanks to the learning process in order to create a better prediction model. The implementation of the technology to develop a sensor for use in real condition environments will then need further studies to determine the right optical waveguide structure to develop, low cost and portable laser sources and spectrometers, as well as the optimized packing and connectors.

## Figures and Tables

**Table 1:** Ge-Se-Te core and cladding layer compositions and optogeometrical properties.

Film	Thickness (μm) ± 0.01 μm	Composition ± 2 at.%	n at 1.55 μm ± 0.01	Δn = n_core_ − n_cladd_
Cladding layer	5.85	Ge_25_Se_61_Te_14_	2.49	0.11
Core layer	5.91	Ge_26_Se_53_Te_21_	2.60	
